# Responsiveness of open innovation to COVID-19 pandemic: The case of data for good

**DOI:** 10.1371/journal.pone.0267100

**Published:** 2022-04-26

**Authors:** Francesco Scotti, Francesco Pierri, Giovanni Bonaccorsi, Andrea Flori

**Affiliations:** 1 Department of Management, Economics and Industrial Engineering, Politecnico di Milano, Milano, Italy; 2 Department of Electronics, Information and Bioengineering, Politecnico di Milano, Milano, Italy; University of Salento, ITALY

## Abstract

Due to the COVID-19 pandemic, countries around the world are facing one of the most severe health and economic crises of recent history and human society is called to figure out effective responses. However, as current measures have not produced valuable solutions, a multidisciplinary and open approach, enabling collaborations across private and public organizations, is crucial to unleash successful contributions against the disease. Indeed, the COVID-19 represents a Grand Challenge to which joint forces and extension of disciplinary boundaries have been recognized as main imperatives. As a consequence, Open Innovation represents a promising solution to provide a fast recovery. In this paper we present a practical application of this approach, showing how knowledge sharing constitutes one of the main drivers to tackle pressing social needs. To demonstrate this, we propose a case study regarding a data sharing initiative promoted by Facebook, the Data For Good program. We leverage a large-scale dataset provided by Facebook to the research community to offer a representation of the evolution of the Italian mobility during the lockdown. We show that this repository allows to capture different patterns of movements on the territory with increasing levels of detail. We integrate this information with Open Data provided by the Lombardy region to illustrate how data sharing can also provide insights for private businesses and local authorities. Finally, we show how to interpret Data For Good initiatives in light of the Open Innovation Framework and discuss the barriers to adoption faced by public administrations regarding these practices.

## Introduction

Countries across the world are facing an unprecedented threat constituted by COVID-19. On January 30^*th*^ 2020, the World Health Organization (WHO) recognized the outbreak as a Public Health Emergency of International Concern (PHEIC) and, as of the end of July 2021, the pathogen is reported to have infected more than 179 million of people with almost 3.9 million of victims at global level. Governments imposed strict policy restrictions on population mobility to mitigate the sharply increasing number of infectious cases, avoiding to overburden the healthcare infrastructures and preserving their capacity to provide adequate assistance to the growing portion of vulnerable population [1]. As a result, the lockdown has kick-started the worst economic recession since the Second World War, with GDP shrinking between 6% and 8% in the Eurozone, US and China [2–4].

Such global emergency represents an example of one of the Grand Challenges to human society, since it is complex to face and poses organizations and countries against an uncertainty that cannot be easily tackled through the reiteration of past solutions [5]. Indeed, it requires the development of novel and original methods based on a multidisciplinary perspective involving a heterogeneous group of collaborative actors [6].

It is against this background that Open Innovation (OI) can be interpreted as a powerful strategy to face such social challenges. Expanding the boundaries of firms and engaging the participation of different stakeholders, OI can in fact contribute to raising the knowledge and the resource bases, thus increasing the likelihood of finding innovative solutions with a positive social impact [7, 8].

OI frameworks enable win-win solutions for public institutions and firms, as the former achieve a positive social impact, while the latter mainly benefit from a more transparent corporate social responsibility (CSR) and from improved reputation [9, 10]. These benefits can be achieved by higher quality projects based on a multidisciplinary approach [11], more dynamic capabilities that support the improvement of firms performances and product innovation [12, 13], and a larger acceptance of the output of the collaboration [14]. More in general, joint problem solving and the exchange of knowledge and material among actors stimulate synergies and create networks that can contribute to generating positive externalities [15–17].

However, the success of these projects is subordinated to an effective management of the main challenges associated to OI [18, 19]. A first source of complexity is represented by the presence of a heterogeneous set of stakeholders with diversified objectives, constituted by policymakers, business entities and end-users [20]. In addition, firms need to find an equilibrium between internal and external OI projects, overcoming the “not-invented-here” syndrome (i.e., the skepticism in relying on solutions developed by other organizations) and the fear to lose the control of their core assets because of the collaboration with partners [21].

 [22] show that the different innovation policies adopted by a set of 44 OECD countries during the COVID-19 crisis can be clustered in 4 groups depending on the extent to which these countries adopted an OI approach interacting with external stakeholders: centralizers, conservative OI promoters, collaborative supporters and open collaborators. In this context a key role was assumed by “ad hoc” task forces involving a combination of experts from universities, research institutions and specific government departments that were assigned to support central governments in taking informed decisions based on the analysis of real data [23, 24]. As a consequence, data sharing and transferring knowledge between governments, laboratories, research centres and private companies became a critical factor in order to provide almost real time evidence on the spread of COVID-19, given the delay in the release of standard official statistics.

With this regard, mobility data describing the movements of individuals in almost real time and with high geographical detail have been widely employed to explain the impact of social restrictions on contagion and human activities [25–29]. Such data have been mainly provided by tech-giants participating in data sharing programs, often called “Data for Good” projects, where large data repositories are shared even with non-profit or public organizations to address the most cutting-edge problems for the society. As examples of the initiatives promoted during the COVID-19 pandemic, Microsoft launched the Open Data Campaign to improve easier and safer data sharing and collaborative networks, Google and Apple published mobility reports to support the study of the pandemic diffusion, and Facebook provided information on social connections and users movements [30–33]. Such initiatives are not only related to the mobility issue, but also provide demographic, economic, environmental and geographical information to help addressing the world’s biggest challenges, thus facilitating a successful collaboration system. Indeed, despite the complexity of integrating massive data coming from different organizations, knowledge sharing is critical to support the actions of public authorities during health emergencies [34, 35]. Furthermore, the aggregation of multi-source data contributes to reduce research and development costs, improving the required time to obtain discoveries, with positive consequences for the whole health community [36, 37].

For these reasons [38], indicates cooperation, openness and data sharing among private and public organizations as imperatives for solving the COVID-19 emergency since this approach could help gathering the global knowledge on this pathogen, enabling faster responsiveness and accelerating the impact of the scientific world to mitigate the pandemic.

In this spirit, our paper distinguishes from extant studies since it attempts to bridge a link between two relevant streams of research, namely the literature on OI aimed to address Grand Challenges and the current research on the usage of almost real time data to support analyses on COVID-19 spread. Our study shows how mobility data disclosed with an open and collaborative approach can be effectively employed to provide scientific evidence on the pandemic dynamics. In particular, we highlight how the OI mechanism can contribute to generating further knowledge to face the pandemic and identify potential solutions in almost real-time. Focusing on the Facebook “Data for Good” initiative, we study how mobility data can be utilized to monitor channels of disease transmission and capture changes in the socio-economic connections in response to governmental restrictive measures implemented in Italy. To this aim, we specifically focus on the period from February 2020 to April 2020 to highlight how mobility data can be employed to study human behaviour during severe restrictions as those related to the lockdown policy. In so doing, we show how mobility information can be integrated with other data sources to interpret in a timely manner the dynamic evolution of users’ habits, constituting a relevant source of information to find out effective solutions. Finally, we discuss how collaboration between private and public organizations can be interpreted within the OI framework; we report some missing elements and barriers occurring during a global emergency, such as the spread of COVID-19, which may limit the potential benefits of OI.

## Literature review

This paper aims to contribute to two wide and recently growing strands of literature: on one side, the research on OI to tackle Grand Challenges, on the other side, the research on the usage of mobility data disclosed by private firms to support scientific evidence on COVID-19.

As to the first research line, OI has been suggested as a powerful strategy to recognize and mitigate the impacts of a relevant shock such as the COVID-19 pandemic. This is because OI involves a coordinated and collaborative effort on complex questions, requiring multidisciplinary contributions and the need to incorporate experts knowledge and perspectives from different fields, leveraging collective wisdom [39]. In a first step the majority of OI initiatives focused on the public health sector, aiming to provide practical solution to face the COVID-19 pandemic [40–42].

In a second step, OI initiatives based on cooperation, collaborative approaches and crowd-sourcing were also undertaken across different domains such as clothing [43], food [44], education [45, 46], capital markets [47] and transportation [48]. For example, in the airway sector [49] describe the case of AirAsia that exploiting organization ambidexterity through an OI approach, identified a good trade-off between exploration and exploitation strategies and reconfigured its business model during the pandemic, boosting revenues.

Although the majority of scientific works focuses on OI benefits during emergency situations at firm level, it is less evident how OI can be exploited by public sector organizations that rarely endorse collaborative innovation models. Indeed, public institutions tend to keep a central governance, lacking a collaborative spirit that is necessary to launch OI strategies [50].

In this sense, the fact that at the COVID-19 outbreak governments designed specific task forces to analyze data and support the decision making process has represented a step toward the adoption of OI practices [22].

The second stream of research we aim to contribute refers to the usage of different types of data related to human movements and interactions to better understand the contagion dynamics and impacts. Several authors have highlighted how the manual reconstruction of the disease transmission chain is not a satisfactory procedure to limit the contagion since it might be hampered by lapses in memory, thus leading to self-reporting biases and unreliable measures [51, 52]. Tracing data obtained via digital apps, instead, has been identified as a powerful tool to overcome these pitfalls and support health authorities to identify adequate mitigation strategies [15, 53]. Indeed, these apps allow to implement social distancing measures, since they speed up the process of identification of face-to-face interactions, enabling the collection of information on proximity contacts that is helpful to advise for medical follow-up and testing [54]. This has been confirmed by several international experiences on digital tracing through app devices, which have shown that it is possible to achieve situational awareness about COVID-19 contagion trends and identify high-risk areas.

For instance, the app “Trace-Together” highlighted the main transmission hotspot in Singapore, discovered in the area of migrant workers dormitories where 78% of the 9,125 confirmed cases were identified at the 22^*nd*^ of April 2020. In South Korea, during the first wave of contagion the exploitation of mobility data, obtained with the tracing app “Corona 100m”, enabled the identification of four main transmission clusters referring to the Shincheonji church, Chungdo Daenam hospital, gym of Cheonam and a pilgrimage tour to Israel [55]. In UK, the usage of the “COVID-19 Symptom Study” mobile application shed light on the geographical distribution of cases and symptoms [56].

Although such data played a key role for managing the early phases of COVID-19 pandemic, there were several shortcomings in their application. First, a key success factor for the effectiveness of this solution was related to the capability to reach a critical mass of users and achieve a high adoption rate. According to the Oxford University’s Big Data Institute, to observe significant results, the digital tool should be rolled out on a large scale, with at least 60% of national population actively using it [57].

Moreover, it became fundamental the development of data protection protocols for privacy and confidentiality safety in order to increase citizens trust on the apps, therefore raising their willingness to accept that a portion of personal mobility data is shared among public and private organizations to face this social challenge [58]. In this sense, several protocols were proposed in 2020 with the objective, on one side, to enable digital contact tracing and reconstruct the virus transmission chain (mostly via Bluetooth technology), and, on the other side, to ensure cybersecurity standards and respect of individual privacy rights with the usage of encrypted random and temporary codes.

However, high privacy requisites had also unwanted consequences for digital tracing apps. First, they slowed down the development process as developers had to obey to higher than usual requirements and policymakers had to supervise the process in short time, often without an available legal and technical framework to assess the quality of the execution [59]. In Europe the majority of apps was ready only by summer of 2020, when the first COVID-19 wave was already extinguished [58]. Second, data from tracing apps could not be shared among different stakeholders [60], therefore, they not only had high development costs but also limited economies of scope.

Given these issues, the best immediate alternative to contact tracing has been identified in anonymized mobility data from mobile phones and social networks [61]. A key advantage of such data is that they were already being collected by companies in accordance with data protection laws and hence immediately shareable with researchers and public organizations. [62] highlighted how mobility data might inform and guide public health actions during COVID-19 life cycle, while a scarce employment of this type of information would translate in missing a relevant opportunity for society to produce insights on the COVID-19 pandemic. For instance [25, 26], exploited data from the Baidu social network to track movements in the province of Wuhan, in [63] data from Cuebiq Data for Good program have been employed to assess the evolution of mobility during lockdown in the United States, while data from Safegraph have been used by [64] to assess the income distribution of isolated individuals in the United States. In Italy, data from Facebook [65] and from Google mobility reports [66] have been exploited to measure changes in the mobility of citizens during the lockdown.

Furthermore, mobility data have a strong potential for impact assessment and the evaluation of policy interventions. Indeed, taking into account mobility data [67], showed that the closure of non-essential activities and the implementation of social distancing involving at least 90% of citizens represent adequate alternatives to limit the contagion. [68] highlighted that mobile phone data representing users movements allowed to demonstrate the effectiveness of mild policies in Sweden to achieve social distancing, as it was reduced by 33% the time spent in work areas and by 38% the average distance travelled per individual. [69] provided a comparison of different interventions based on mobility data, showing that to avoid the resurgence of the contagion multiple non pharmaceutical interventions are required until vaccine supply.

In addition to this, mobility data have also been used to identify environments that facilitate virus spreading such as shopping malls, sport facilities, leisure centres, public libraries, theatres and cinemas, clarifying also how short and long distance travelling may contribute to the spread of the contagion [70, 71]. Furthermore, the integration of mobility and epidemiological data have been widely used in order to explain or predict COVID-19 cases with high levels of precision, highlighting how different economic sectors impact on the virus diffusion [28, 29, 72]. In general, mobility data have been suggested as a relevant input information to support the design of optimal restriction strategies [73, 74]. Indeed, different authors highlighted how generalized national lockdown measures disproportionately affected local territories, disrupting existing value chains and raising inequalities across areas [27, 65, 75, 76].

In the next sections, we will use data from Facebook “Data for Good” initiative as case study to illustrate how mobility data can be employed to the study of COVID-19 impacts in different scenarios during crisis periods. By doing so, we are not suggesting that mobility data should replace tracing apps, as they are tailored to different and complementary needs. We instead stress the case that such aggregated mobility data should be sought by public organizations as first response tools, due to their availability and potential integration with other data sources.

## Data and methodology

We analyze mobility in the Italian peninsula based on movement maps provided by Facebook to the research community through its “Data for Good” program [77], as a demonstrative example of how knowledge sharing between private and public organizations can fuel OI mechanisms that can be exploited to face social challenges [78]. These movement maps consist of de-identified and aggregated information on Facebook users leaving their geo-positioning system enabled, representing movements across Bing tiles and administrative units (e.g., Italian municipalities). The data collection pipeline of Facebook builds upon the Bing tile gridding of the earth’s surface, developed by the online cartographic platform operated under the Bing division of Microsoft. Similar to data analyzed in similar works on mobility restrictions [25, 26, 65], these measurements do not represent the actual number of individuals traveling. Rather, they provide an index of mobility, constructed by Facebook with proprietary methods to ensure privacy protection [61], which correlates well with real movements of people [77].

In our study, we rely on data at the municipality level to analyze national mobility patterns, while to investigate peculiarities of each zone within a given municipality we refer to a more granular spatial level corresponding to mobility within city tiles of size 0.3km by 0.3km. Data referring to movements between municipalities were collected from February 23^*rd*^ 2020 to April 12^*th*^ 2020, with about 1 million distinct observations (with 8-hour frequency) covering approximately half of the entire number of Italian municipalities. For what concerns mobility flows in the metropolitan area of Milan (which correspond to a higher resolution w.r.t movements between municipalities), Facebook released data starting from April 6^*th*^ 2020. We collected these flows for about 3 weeks until April 26^*th*^ 2020. During the observation period, the average number of daily Facebook users in Italy with their location enabled was approximately 4 million.

Finally, our work relies on the use of a network science approach to analyze mobility based on graph formalism. A graph is a collection of vertices (or nodes), which represent entities, and arcs (or edges), which represent existing relationships between entities. A graph can be directed if links are oriented, un-directed otherwise, and weighted if each link is associated to a distinct value (or weight). A link starting and ending on the same vertex is called self-loop. In particular, we represent networks of mobility as weighted directed graphs where nodes are municipalities (or tiles, smaller areas inside municipalities) and edges are weighted based on the amount of traffic from source to destination. We define the strength of a node as the sum of weights over in-going and out-going edges, excluding self-loops, i.e. edges sharing the same source and target nodes, which are analyzed separately. This representation allows us to describe both traffic flowing between locations (using the strength) and the internal mobility of a specific location (using the self-loop).

Furthermore, we build mobility networks at province and regional level by aggregating over the municipalities belonging to them, excluding their self-loops: this means that self-loops of provinces and regions represent movements between different municipalities of the same aggregate administrative territory (without capturing their internal mobility).

## Results

In the outbreak of COVID-19 pandemic, social distancing has been seen as the solution to mitigate the spread of contagion. As a consequence, recent research has focused on human mobility to understand the extent to which people are adhering to governmental lockdown restrictions [63, 79–81].

To highlight how population reacted to policy restrictions, we employ an illustrative study at different scales of analysis. First, we show how information embedded in human mobility can be used to reveal changes both at short and long distances. This aspect is of utmost relevance for policymakers, revealing the emergence of potential channels of contagion across different geographical areas, and for business reasons since it indicates the segregation of economic activities. Second, we show how such mobility flows can be investigated to depict the network of connections in a local context, such as an Italian province.

Finally, we provide a micro-level analysis focused on urban mobility in the city of Milan. Following literature that proposes the use of mobile applications to map mobility and people concentration for the study of the spreading of contagion [61, 79, 80], we show how lockdown measures affected in a heterogeneous way the flow of mobility across distinct city districts. In particular, we analyze two cases regarding the lockdown period: first, we focus on differences in mobility for those city areas where at least one mass market retailer (MMR) or a large food retailer is located; second, we repeat a similar analysis but focusing on the location of hospitals. We observe how aggregated flows of mobility might support not only more responsive actions to contain the pandemic, but also be exploited to reshape business activities and support policy decisions.

### Mobility patterns under different aggregation scales

**Fig 1:**
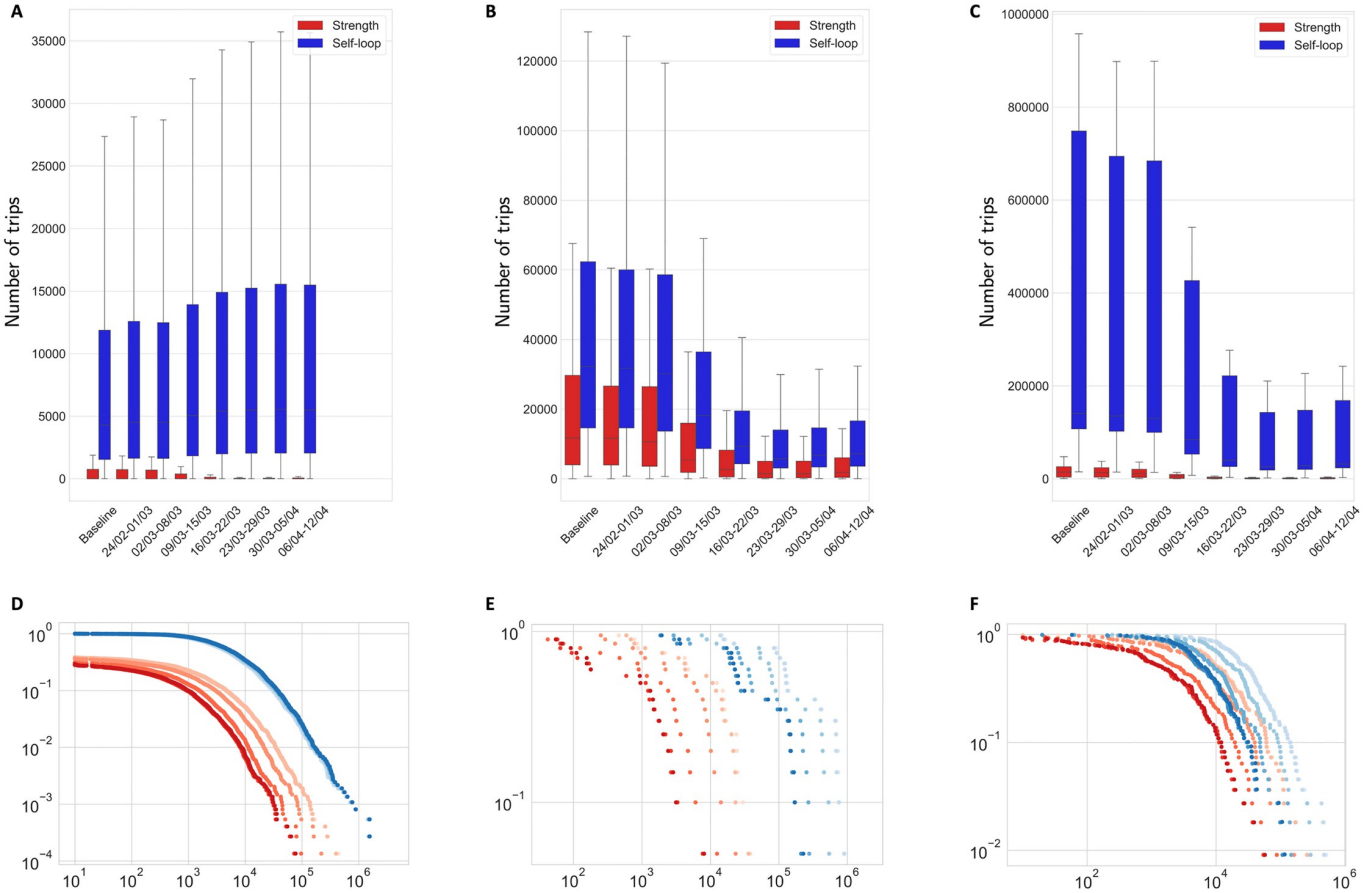
Mobility flows distribution at municipal, province and regional level. (A) and (D) show flows at municipal level, (B) and (E) show flows at province level and (C) and (F) show flows at regional level. Red indicates flows across administrative units, while blue stands for inner self-loops. In the top row we also show values for the “baseline” mobility network, which is built considering the average of daily observations over a window of 45 days prior to February 23rd. The bottom row shows the distribution of values for each week using a color scale that maps lighter colors to earlier periods. Application of one way ANOVA test between distributions of indicators in the 2nd and 3rd weeks (which capture the transition to national closure) are: Municipalities: Strength (Statistic = 27.50, PVAL ~ 0), Self-Loops (Statistic = 0.488, PVAL = 0.485); Provinces: Strength (Statistic = 3.92, PVAL = 0.049), Self-Loops (Statistic = 4.40, PVAL = 0.037); Regions: Strength (Statistic = 3.30, PVAL = 0.077), Self-Loops (Statistic = 1.50, PVAL = 0.223).

Italian mobility has been profoundly affected by policy measures aimed to mitigate the initial spread of contagion. Such reductions in mobility patterns clearly emerge at different levels of analysis. Fig 1 shows the distribution of mobility between regions, provinces and municipalities, both in term of outgoing links (red bars) and inner self-loops (blue bars).

Regarding the strength of links connecting different administrative units, it can be seen that the deployment of lockdown restrictions on March 8^*th*^ − 9^*th*^ 2020 [82, 83] deeply reduced the connectivity at all geographical levels. In particular, ANOVA one way tests between the second and third week of the analyzed timeframe (02/03–08/03 with respect to 09/03–15/03) clearly reject the null hypothesis of no difference in the mean number of trips between the two periods, with p-values equal to ∼ 0, 0.049 and 0.077 for municipalities, provinces and regions, respectively. This appears coherent with the governmental effort to circumscribe the diffusion of contagion by preventing flows of people moving from the most negatively affected territories of Northern Italy to the rest of the country.

We observe heterogeneous patterns at different levels of geographical granularity for what concerns inner loops. At municipality level, inner connectivity does not seem to decrease during the lockdown, which can be explained by work reasons. Indeed, an ANOVA one way test does not reject the null hypothesis of absence of difference in the mean number of trips at municipal level between the pre-lockdown and the first lockdown week (p-value equal to 0.485). Conversely, we highlight a significant contraction of inner mobility at province level (p-value equal to 0.037). At regional scale, the difference in the average number of trips between the pre-lockdown and the lockdown period becomes significant starting from the second week of restrictions (for the first week of lockdown the p-value is 0.223). More in general, these mobility patterns suggest how competing behaviors might emerge with respect to the distance of the movement. Lockdown restrictions appear, in fact, more effective in reducing medium-long transfers, thus contributing to the isolation of large geographical areas, while local mobility appears less affected and more prone to maintain a more stable amount of connections.

Although mobility flows reported in Fig 1 provide supporting evidence that policy restrictions reduced the overall degree of connection of the Italian mobility network, still a certain level of heterogeneity seems to emerge once we move from national to the local level of analysis. For this reason, we select the case of the Milan province as an illustrative example of mobility network features before and after the deployment of lockdown measures.

**Fig 2:**
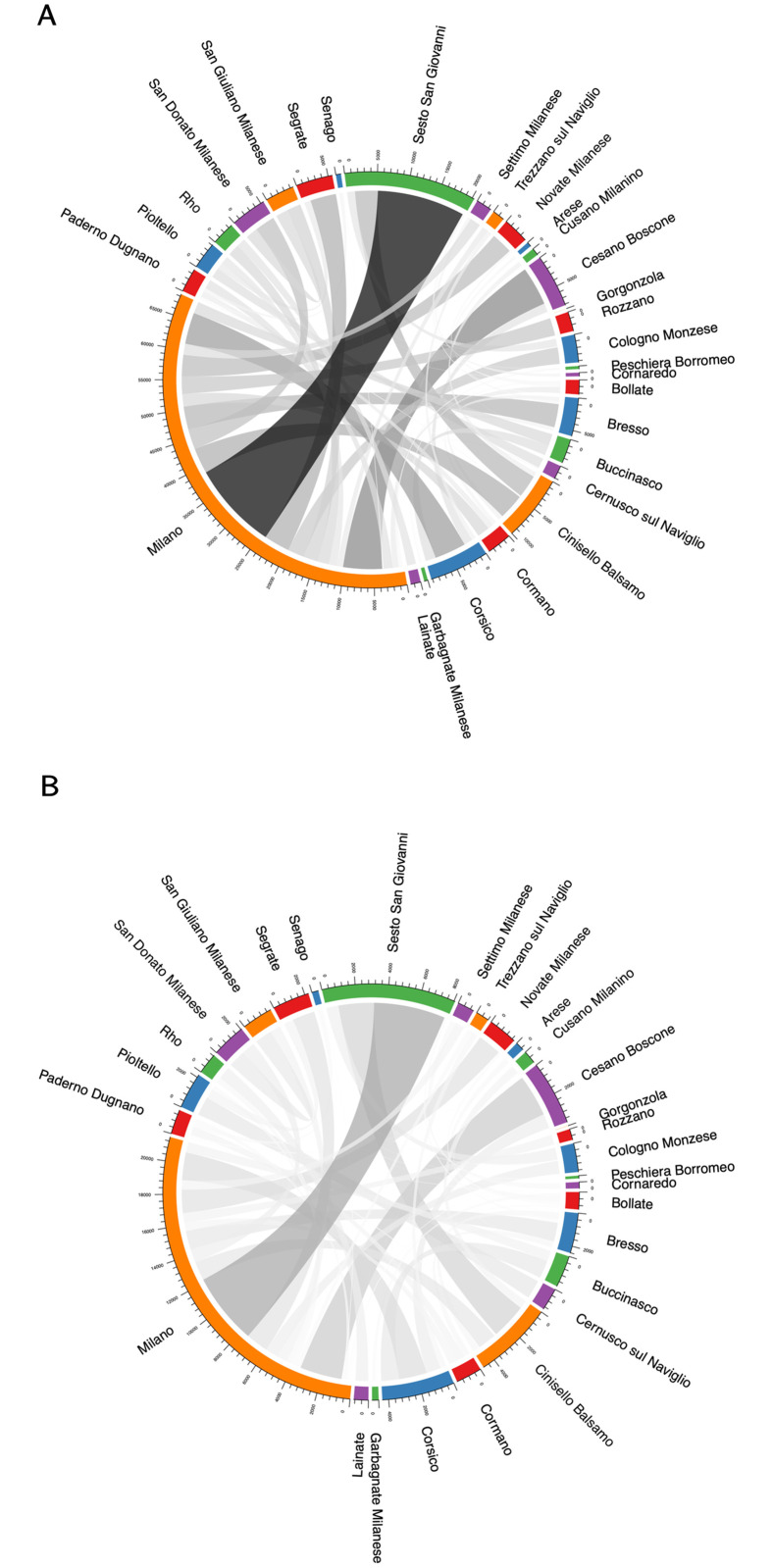
Mobility flows in the province of Milan. (A) and (B) show the mobility between municipalities in Milan province before (top) and during (bottom) lockdown, respectively. Color intensity indicates the amount of traffic (with darker edges corresponding to higher values), and the same normalization is used across the two diagrams. Properties of the mobility network [before, during] lockdown: Assortativity: [-0.062, -0.063]; Average Clustering Coefficient: [6.28e-05; 2.70e-05].

Fig 2 shows the strength of un-directed connections (i.e. we sum the traffic in both directions) among municipalities composing the Milan province (we only show the first 30 municipalities in terms of population), with larger and darker color lines referring to the strongest connections and thinner and lighter colors to weak connections.

As shown in Fig 2, Milan plays a pivotal role in both periods, being very central both in the amount of incoming and outgoing links. This topological position is maintained during the lockdown phase and, similarly, other municipalities such as Cinisello Balsamo, Cesano Boscone, Corsico and Sesto San Giovanni, remain at the core of the network while the overall connectivity translated to lower values. More specifically, we compare the distribution of the daily values of assortativity and average clustering coefficient of the network in the two weeks before the lockdown and in the first two weeks of national restrictions, coherently with the time period analyzed in Fig 2. We show that the topological structure of the network is not subject to relevant changes in terms of assortativity, as a Kruskal-Wallis test on the two distributions does not reject the null hypothesis that the two samples are extracted from the same population (p-value = 0.613). Conversely, the average clustering coefficient exhibits statistically significant differences (p-value = 0.015) suggesting that lockdown measures contributed to a fragmentation of the mobility network with a relevant reduction of connections across territories. This result indicates that the emerging mobility dynamics are affected by the deployment of lockdown restrictions, whose main result seems to be related not only to the sharp fall in the aggregate amount of flows but also to some changes in the topological properties of the network configuration.

Furthermore, as shown in Fig 1, lockdown measures actually limit movements across administrative units but are likely to increase inner mobility, especially for small towns, as individuals are forced to comply with lockdown measures. At municipality level, the Pearson correlation between the variation in inner mobility during the deployment of lockdown and the corresponding population is −0.63 (p-value = 0.0001), while the correlation with population density is −0.51 (p-value = 0.003).

**Fig 3:**
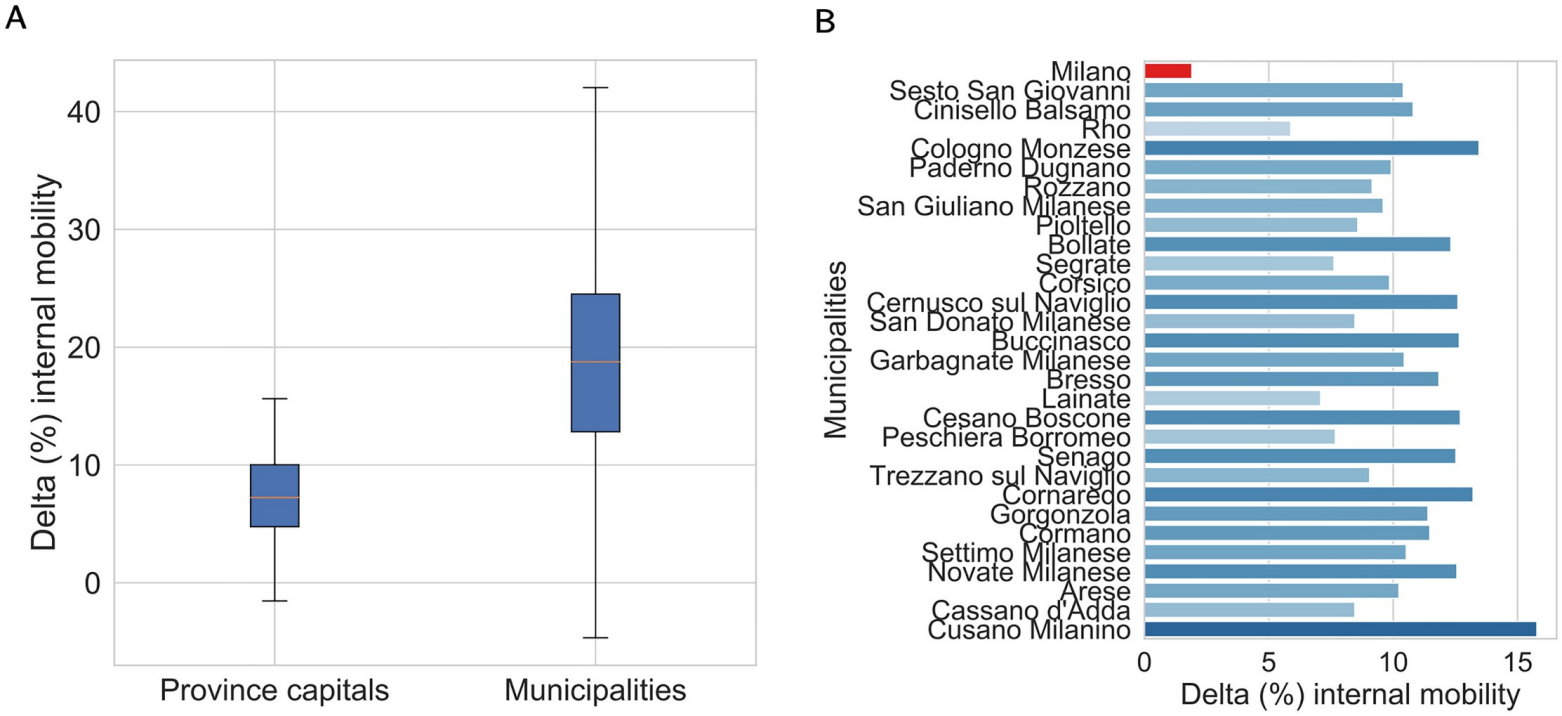
Percentage variation of internal mobility for municipalities between the periods during and before lockdown. (A) compares the distribution for municipalities and province capitals in the entire peninsula, whereas (B) shows a detailed overview on Milan province and its municipalities.

Such patterns can be explored more in detail by focusing on each municipality composing a certain province. Fig 3 shows how the internal mobility in the city of Milan during the lockdown phase increases less than in other municipalities of the province, clearly suffering from the absence of commuting (and in general of non-residents who returned to their home towns), which most likely contribute to the level of mobility of the province capital in business as usual days.

More generally, such findings emerging from mobility networks at local level may help to disentangle possible changes in mobility patterns that arise due to the interplay between policy restrictions and people reactions. These effects in terms of variations in network properties may be included in the decision processes of both policymakers and business firms when designing appropriate actions to respond to emerging mobility needs, possibly captured by integrating different data sources. We propose some examples in the following subsection.

### Mobility patterns and local features

In this section, we explore some drivers at the intersection between changes in urban mobility and city features. We focus again on the Milan administrative unit, but in this case we consider the municipality level. We enrich mobility data with additional layers of analysis corresponding to the geolocalization of MMRs, large food retailers and hospitals disclosed by the Open Data website of Lombardia region [84], thus providing alternative paradigms to describe potential flows of people during the lockdown phase.

**Fig 4:**
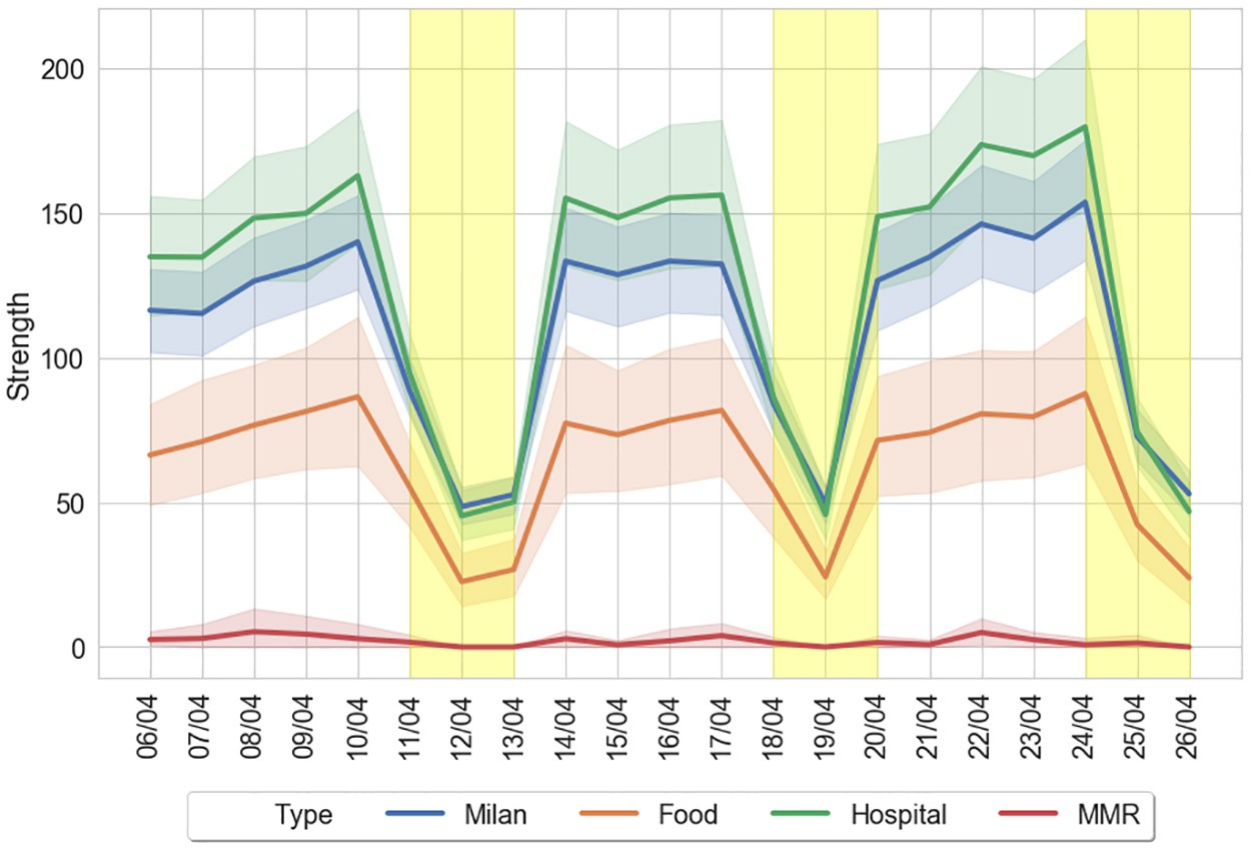
Milan mobility flows. We partition the city of Milan into Bing tiles of size 0.3km by 0.3km. We label each tile as: MMR (in red), Food (in orange) or Hospital (in green) if at least one of the corresponding places of interest is located. Such categorizations are mutually exclusive, meaning that if in the same tile more than one category is present than this tile is not assigned to a specific category. For each category we show the mean value and 95% C.I. with shaded areas; Milan city tiles considered altogether are shown in blue color. Yellow areas indicate weekends and holidays.

Basically, we inspect all Bing tiles in Milan and we differentiate between those having at least one MMR shop, a large food retailer, or a Hospital from the other districts of the city. We consider as large food retailers those medium-large shops whose food-related area is more than 50%. We take into account only disjoint tiles, meaning that we focus specifically on those city districts where only one of the aforementioned categories of place of interest is located. In so doing we are able to distinguish between city districts characterized by the presence of at least one MMR, one food-related shop such as a supermarket, or a hospital, but not with the presence of more than one of these types of place of interest, thus hopefully depicting cleaner differences in the emerging patterns of mobility. Then, we study the strength of the connections of these sub-portions of the city for which at least one place of interest is located w.r.t the city altogether. Fig 4 shows the daily mobility trend for these districts during a 3-week period in April 2020.

Some interesting results clearly emerge. First, districts hosting Hospitals are more likely to intercept a larger flow of mobility compared to the rest of the city probably due to the pandemic outbreak at that period. Second, Food-related districts are less central than average, but still more popular than those areas characterized by MMRs only. Third, notwithstanding the policy restrictions, still the week-end effect distinctly characterizes the decrease of mobility, thus suggesting that during the week a non-negligible portion of inhabitants continues to move, probably for work reasons. Fourth, such mobility patterns are able to capture specific events, such as the closure of shops in Easter Monday (13^*th*^ April 2020).

Despite the aggregate provision of data, these patterns thus seem to be able to highlight relevant behaviors during the lockdown phase, constituting an interest instrument for both policy regulators and firms to manage distressed scenarios as those related to the evolution of the COVID pandemic.

### The innovation value of mobility data

Openness unleashes a rapid response against COVID-19 pandemic as scientists, policymakers and business firms are mobilized to cooperate to figure out effective solutions. This attitude corresponds to what in literature has been recognized as OI [85]. Our previous examples illustrate how “Data for Good” initiatives can provide relevant information to the research community, suggesting how heterogeneous entities and forces are required to cope with complex social challenges [86]. Indeed, the OI approach is critical during such emergencies as it accelerates the process of knowledge collection and speeds up the time to market of potentially effective solutions [87].

The innovation mechanism provided by “Data for Good” initiatives to face social challenges relies on the less common outbound (or “inside-out”) OI as the actors canalize the innovation into external markets or organizations that better suit its diffusion and exploitation [11, 88, 89]. Our analysis has highlighted some of the benefits that might arise from the adoption of this approach by exhibiting the role of mobility data in order to highlight the effects of lockdown on connectivity and on changes in human behaviours.

Nevertheless, mobility data might be used not only to assess the impact of policy makers decisions and identify potentially risk areas, but they might be also employed to interpret how changes in the habits of individuals affect future business scenarios. For example, the higher growth of the population mobility during the reference period for Italian small municipalities with respect to province capitals sheds lights on adjustment mechanisms of population concentration. In particular, this pattern might have consequences on the redistribution of expenditures and consumption across geographical areas, with large urban centres that are expected to be the most penalized sites. Firms with many points of sale along the Italian peninsula might be affected for instance by a geographical redistribution of their turnover, with establishments in big metropolitan areas reducing their market share in the company portfolio in favor of those in smaller areas. In addition, lower mobility levels across different municipalities, provinces and regions can have a negative impact on the number of customers physically reaching stores. As a consequence, companies could reflect upon the possibility to proactively rearrange their business models to enable a multi-channel value proposition to reach purchasers both through e-commerce platforms and physical stores. Shared data may provide inspiration for new or more effective solutions to already existing firms’ problems bypassing the not-invented-here syndrome. More generally, such data sharing initiatives may contribute to the analysis of socio-economic dimensions in almost real time. Finally, publicly available data may act as solution of last resort for unforeseen problems, for instance during humanitarian emergencies when usual information channels are less reliable (see, e.g., [77] for the use of social network data during natural disasters).

Our analysis has shown that the share of data between a social network and the research community can serve both societal and business interests and aims to be an illustration of the benefits that this type of public-private cooperation might produce in tackling a Grand Challenge, such as the COVID-19 pandemic. In this sense, by exhibiting some insights that can be captured through the exploitation of mobility data, we have tried to formalize the advantages that can be learnt from this type of experience. Next section frames this approach within the OI framework and discusses some barriers that may have hampered its widespread practical application, highlighting some factors that should be addressed and transformed by society to effectively capture the potential advantages.

## Conclusions

With data sharing firms allow external actors to access their private data without being part of the organization and, often, without having any form of partnership with the data owner.

As a form of collaboration between public and private organizations, data sharing belongs to the OI framework: to use the taxonomy outlined in [90] it can be considered as outbound and non-pecuniary OI, also described as “collective invention”. Collective invention has been recognized as an optimal innovation strategy when the scope of cooperation invests social challenges and community issues, transcending the usual business and technological boundaries of firms [91]. Indeed, it reduces the cost to access geographically dispersed knowledge [92], allows larger innovation diffusion [93, 94] and improves the reputation of the companies involved [95, 96].

However, unlike previous experiences in the healthcare sector (cfr. the Innocentive and the Harvard Catalyst examples [97]), data sharing initiatives are usually tailored toward the non-profit community. This is because, on one side, researchers and ONGs are expected to better understand and exploit the value of data and, on the other side, they do not represent competitors for the sharing organization. Accordingly, the “Data For Good” initiatives launched during the COVID-19 pandemic are explicitly designed to provide support for humanitarian purposes.

Such data sharing can be better identified as a form of corporate social responsibility [98, 99]. In fact, the type of framework adopted by data sharing organizations does not correspond to an Open Innovation approach: they do not foresee specific partners interacting with the technology (mobility data in our case), owning and modifying it. This aspect does not prevent data sharing firms from playing the role of innovation intermediaries for the mobility data technology, to the extent they are coordinating a market—such as public administrations looking for an innovative solution to a complex problem—with solution providers such as tech firms. Moreover, given the non-pecuniary nature of their involvement, they can also be associated with the role of innovation missionaries: actors inside the innovation process whose aim is to diffuse a certain technology since they believe it is beneficial for the society [100].

The duality in the nature of “Data For Good” activities, closed-innovation solutions to open problems, highlights that there are still some barriers to the adoption of the mobility data technology and to the achievement of potential advantages of an OI approach. The fear of loosing competitive advantages by private firms [62] may shrink their willingness to exchange knowledge. Moreover other sector specific factors hurdle the diffusion of innovations in public administrations [101, 102]: often cited reasons are a risk-adverse culture, a hierarchical bureaucratic organization and a lack of coordination among central and local governments [103].

However, the emergence of crises at local and global level allows to overcome these barriers since *“in those cases public awareness, media and political support create an environment in which risk taking is legitimized, leadership and funding is made available and experimentation possible”* [103]. This has been the case for the development of open data protocols for health reports during the Ebola virus crisis [34, 104], which resulted in an efficient implementation during the current COVID-19 pandemic [35, 105].

Unfortunately, a similar approach has not been extended to mobility data [61, 62]. Further investigation will be required to assess the precise reasons why the COVID-19 crisis has not led public institutions to develop open data protocols regarding mobility information. In Europe a possible explanation may be related to the recent European legislation on data protection, the General Data Protection Regulation (GDPR) [106]. By increasing the legal requirements for data collection, the legislation may have increased the transaction costs for firms collecting mobility data, slowing down the development of publicly available datasets. This is also suggested by previous surveys regarding Open Innovation practices in public governance [101], which report that inadequate legislation is a contextual barrier for innovation in public governance 66,6% of the times.

During critical times, citizens, firms and governments are often called to provide innovative solutions to challenging problems. Anonymized mobility data allow organizations to reach a great depth of analysis without relying on user effort (or donation), by simply leveraging data collected during the standard use through a law compliant procedure. In the spirit of OI, this technology is not only already available, but it has also been shared with the research community to help tackle the diffusion of the contagion. One technology, anonymized mobility data, can thus be employed to address several issues of public interest.
